# Omega‐3 polyunsaturated fatty acid supplementation reduces blood pressure but not renal vasoconstrictor response to orthostatic stress in healthy older adults

**DOI:** 10.14814/phy2.13674

**Published:** 2018-04-19

**Authors:** Christine M. Clark, Kevin D. Monahan, Rachel C. Drew

**Affiliations:** ^1^ Penn State College of Medicine Milton S. Hershey Medical Center Hershey Pennsylvania; ^2^ Penn State Heart and Vascular Institute Penn State College of Medicine Milton S. Hershey Medical Center Hershey Pennsylvania

**Keywords:** Aging, blood pressure, fish oil, orthostasis, renal vasoconstriction

## Abstract

Older adults exhibit augmented renal vasoconstriction during orthostatic stress compared to young adults. Consumption of omega‐3 polyunsaturated fatty acids, such as eicosapentaenoic acid (EPA) and docosahexaenoic acid (DHA) found in fish oil (FO), modulates autonomic nerve activity. However, the effect of omega‐3 polyunsaturated fatty acid consumption on the renal vasoconstrictor response to orthostatic stress in young and older adults is unknown. Therefore, 10 young (25 ± 1 years; mean ± SEM) and 10 older (66 ± 2 years) healthy adults ingested 4 g FO daily for 12 weeks, and underwent graded lower body negative pressure (LBNP; −15 and −30 mmHg) pre‐ and post‐FO supplementation. Renal blood flow velocity (RBFV; Doppler ultrasound), arterial blood pressure (BP; photoplethysmographic finger cuff), and heart rate (electrocardiogram) were recorded. Renal vascular resistance (RVR), an index of renal vasoconstriction, was calculated as mean BP/RBFV. All baseline cardiovascular values were similar between groups and visits, except diastolic BP was higher in the older group (*P *<* *0.05). FO supplementation increased erythrocyte EPA and DHA content in both groups (*P *<* *0.05). FO did not affect RVR or RBFV responses to LBNP in either group, but attenuated the mean BP response to LBNP in the older group (older −30 mmHg: pre‐FO −4 ± 1 vs. post‐FO 0 ± 1 mmHg, *P *<* *0.05; young −30 mmHg: pre‐FO −5 ± 1 vs. post‐FO −5 ± 2 mmHg). In conclusion, FO supplementation attenuates the mean BP response but does not affect the renal vasoconstrictor response to orthostatic stress in older adults.

## Introduction

Aging is associated with a greater incidence of orthostatic hypotension (Rutan et al. 1992), which is linked with greater mortality (Masaki et al. 1998). Maintenance of adequate blood pressure (BP) is determined by several reflex neural mechanisms, primarily the baroreflex, which regulates second‐to‐second BP (Mark and Mancia 1983). In response to a drop or rise in BP, the baroreflex can increase or decrease sympathetic nervous system activity, respectively, to correct and return BP to its original level (Mancia and Mark 1983). Sympathetic neural outflow from the medulla oblongata in the brainstem directed towards the kidneys induces renal vasoconstriction, which reduces renal blood flow (Johns et al. 2011). This reflex reduction in renal blood flow allows blood to be redistributed to other areas of the body where it is needed more critically at that time, such as the systemic circulation to maintain BP. The kidneys receive ~25% of resting cardiac output (Zelis et al. 1988), so they serve an integral role in systemic BP regulation. Advancing age is linked with numerous changes in physiological function related to BP control, including increased sympathetic neural outflow (Sundlöf and Wallin 1978), greater arterial stiffness (Mitchell et al. 2004), and decreased baroreflex sensitivity (Gribbin et al. 1971). We recently showed that the sympathetically mediated renal vasoconstrictor response to lower‐body negative pressure (LBNP), an experimental approach that simulates orthostatic stress, is augmented in healthy older adults, while their BP response is similar to healthy young adults (Clark et al. 2015). Finding interventions to improve cardiovascular responses to orthostatic stress in older adults is critically important, given the higher frequency with which this population experiences orthostatic hypotension (Rutan et al. 1992), and the associated greater mortality (Masaki et al. 1998). Furthermore, exaggerated renal vasoconstriction in response to orthostatic stress in older individuals may be related to the decline in renal function linked with advancing age (Weinstein and Anderson 2010), due to repeated greater reductions in renal blood flow over time, as well as an increased risk of chronic kidney disease (Franceschini et al. 2010). Therefore, discovering interventions that reduce this enhanced renal vasoconstriction, while maintaining or preferably improving the BP response to orthostatic stress, is also important to potentially lessen the cumulative negative impact on the kidneys in older adults.

Consumption of omega‐3 polyunsaturated fatty acids, such as eicosapentaenoic acid (EPA) and docosahexaenoic acid (DHA) found in fish oil (FO), is associated with lower cardiovascular mortality (GISSI‐Prevenzione Investigators, 1999; Predimed Investigators, 2016). We recently showed in healthy older adults that FO supplementation decreases central arterial stiffness (Monahan et al. 2015), an important prognostic indicator of future cardiovascular events and all‐cause mortality (Vlachopoulos et al. 2010). In older adults, consumption of baked or broiled fish can lower the risk of developing congestive heart failure (Mozaffarian et al. 2005), a condition associated with increased sympathetic activation. The effect of FO consumption on neurally mediated cardiovascular responses to physiological stressors has been examined in some previous studies, with varying results. FO supplementation has been shown to attenuate total muscle sympathetic nerve activity and heart rate (HR) responses to mental stress (Carter et al. 2013), yet augment muscle sympathetic nerve activity responses to fatiguing ischemic handgrip exercise and a cold pressor test (Monahan et al. 2004), reduce the forearm vasoconstrictor response to intra‐arterial infusion of norepinephrine in healthy young men (Chin et al. 1993) and overweight young‐to‐middle‐aged men (Mori et al. 2000), and improve baroreflex‐mediated responses to hypertensive stimuli in patients with heart failure (Radaelli et al. 2006). Few investigations have involved assessing the effect of FO consumption on responses to orthostatic stress, or the effect of age on these responses. Mills et al. (1990) showed that FO supplementation did not affect the increase in forearm vascular resistance in response to LBNP in healthy young men. We recently showed that FO supplementation attenuates the mean and diastolic BP increases at the onset of isometric handgrip exercise, a time of rapid reflex autonomic adjustments, in both healthy older and young adults (Clark et al. 2016). However, the effect of FO consumption on the sympathetically mediated renal vasoconstrictor response to orthostatic stress in older adults has not been determined. Given the importance of finding potential therapies to improve orthostatic responses in older individuals, particularly their augmented renal vasoconstrictor response, this area of research warrants investigation.

Accordingly, we examined this concept by assessing the renal vasoconstrictor response to orthostatic stress via LBNP before and after 12 weeks of FO supplementation in healthy older and young adults. As older adults exhibit an augmented renal vasoconstrictor response to LBNP (Clark et al. 2015), and FO supplementation can attenuate sympathetic responses (Chin et al. 1993; Mori et al. 2000; Radaelli et al. 2006; Carter et al. 2013), we hypothesized that FO supplementation would reduce the greater renal vasoconstrictor response to LBNP in healthy older adults. Findings from this investigation would provide clinically relevant insight into the effect of omega‐3 polyunsaturated fatty acid consumption on the age‐related renal vasoconstrictor response to orthostatic stress.

## Methods

### Ethical approval

The experimental protocol received approval from the Institutional Review Board at the Penn State Milton S. Hershey Medical Center and conformed with the Declaration of Helsinki. The studies were performed in the Clinical Research Center of the Penn State Milton S. Hershey Medical Center. The purpose of the study and risks involved were explained to all subjects, and written informed consent was obtained.

## Subjects

Ten young and ten older healthy subjects participated in this study (Table 1). All subjects met the following inclusion criteria: normotensive, nonsmokers, no history of cardiovascular or renal disease, not taking any medications that could impact autonomic or cardiovascular function, were recreationally active with at least 2–3 h of aerobic exercise per week, and were in good health as assessed by history and physical examination. Female subjects of childbearing age had a urine pregnancy test to rule out pregnancy. All subjects were asked to refrain from performing strenuous exercise, ingesting caffeine and alcohol for 24 h, and ingesting food for 8 h prior to their study visits. Blood samples were obtained for comprehensive metabolic panel and complete blood count screening, including measurement of baseline plasma creatinine and blood urea nitrogen to assess renal function pre‐and post‐FO supplementation (Clinical Laboratories, Penn State Milton S. Hershey Medical Center), from all subjects pre‐ and post‐FO supplementation except one older subject post‐FO supplementation.

**Table 1:** Baseline characteristics, cardiovascular, and eicosapentaenoic acid (EPA) and docosahexaenoic acid (DHA) content values pre‐ and post fish oil supplementation for the young and older groups

	Young, pre‐fish oil	Young, post‐fish oil	Older, pre‐fish oil	Older, post‐fish oil
Number of subjects (men/women)	10 (5/5)		10 (3/7)	
Age (y)	25 ± 1		66 ± 2*	
Height (m)	1.78 ± 0.02	1.78 ± 0.02	1.68 ± 0.03*	1.69 ± 0.03*
Weight (kg)	82 ± 4	82 ± 4	69 ± 4*	69 ± 4*
RVR (au)	1.69 ± 0.09	1.85 ± 0.19	2.00 ± 0.24	2.01 ± 0.27
RBFV (cm/sec)	52.6 ± 2.8	50.2 ± 4.2	51.0 ± 5.7	52.1 ± 6.1
MAP (mmHg)	87 ± 1	86 ± 1	91 ± 2	91 ± 2
SBP (mmHg)	118 ± 3	118 ± 3	125 ± 3	124 ± 4
DBP (mmHg)	66 ± 2	66 ± 1	72 ± 1*	71 ± 2*
PP (mmHg)	52 ± 2	52 ± 3	53 ± 3	53 ± 4
HR (b/min)	58 ± 2	57 ± 3	63 ± 3	61 ± 3
Erythrocyte EPA content (%)	0.50 ± 0.03	1.86 ± 0.48†	0.57 ± 0.07	3.22 ± 0.56†
Erythrocyte DHA content (%)	4.27 ± 0.16	6.02 ± 0.61†	3.60 ± 0.33	6.97 ± 0.62†
Plasma creatinine (mg/dL)	0.81 ± 0.03	0.80 ± 0.03	0.85 ± 0.03	0.84 ± 0.03
Plasma blood urea nitrogen (mg/dL)	12.8 ± 1.1	13.3 ± 1.2	15.2 ± 0.9	16.0 ± 1.2

Data are shown as mean ± SEM. All values are for *n *=* *10 young and *n *=* *10 older subjects, except EHA and DHA content values that are for *n *=* *7 young and *n *=* *8 older subjects, and post‐fish oil creatinine and blood urea nitrogen that is *n *=* *10 young and *n *=* *9 older subjects. DBP, diastolic blood pressure; HR, heart rate; MAP, mean arterial blood pressure; PP, pulse pressure; RBFV, renal blood flow velocity; RVR, renal vascular resistance; SBP, systolic blood pressure

*Significantly different from young (*P *<* *0.05). ^†^Significantly different from pre‐fish oil.

Data on renal, BP, and HR responses to LBNP without FO supplementation from all 10 young and 10 older subjects have been previously published in our paper in which the effect of aging on the renal vasoconstrictor response to orthostatic stress in healthy adults was examined (Clark et al. 2015). Additionally, data on erythrocyte EPA and DHA content values pre‐ and post‐FO supplementation from 7 of the 10 young subjects and 5 of the 10 older subjects have been previously published in our paper in which the effect of omega‐3 polyunsaturated fatty acid consumption on age‐related cardiovascular responses at the onset of isometric handgrip exercise was examined (Clark et al. 2016). The focus of this study is the effect of omega‐3 polyunsaturated fatty acid consumption on the renal vasoconstrictor response to orthostatic stress in healthy young and older adults, which has not been investigated to date. Novel data on renal, BP, and HR responses to LBNP following FO supplementation in all 10 young and 10 older subjects are presented here. Renal, BP, and HR responses to LBNP prior to FO supplementation are included to serve as a baseline pre‐FO comparison, and EPA and DHA content values pre‐ and post‐FO supplementation are included to illustrate the effectiveness of the FO intervention in increasing EPA and DHA levels in the subset of subjects involved in this study.

### Intervention

**Figure 1:**
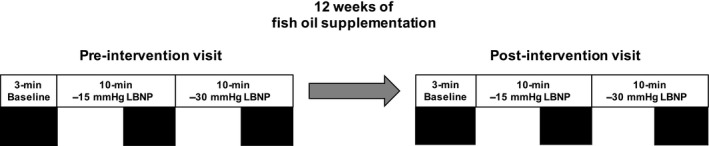
Schematic depiction of the experimental protocol and timeline of the intervention. Shaded area denotes 3‐min periods used for data analysis. LBNP, lower‐body negative pressure.

Subjects underwent pre‐ and post‐intervention visits in relation to their FO treatment (Fig. 1). After completing the procedures and measurements in the pre‐intervention visit, subjects began daily ingestion of 4 g of highly purified and concentrated FO supplements (omega‐3 acid ethyl esters; Lovaza; GlaxoSmithKline, London, UK) for 12 weeks. FO supplementation consisted of 4 × 1 g capsules, each containing at least 900 mg of the active ingredients EPA and DHA. Compliance was assessed by use of pill diaries, pill counts, and phone calls from investigators, and erythrocyte EPA and DHA levels were quantified in a subset of subjects (see “EPA and DHA quantification” section below). Subjects then returned for a post‐intervention visit, which involved the same procedures and measurements as the pre‐intervention visit.

### Experimental protocol

Subjects lay supine, encased up to the level of the iliac crest in an LBNP chamber, and were then instrumented. After at least 30 min, a 3‐min baseline period occurred before LBNP was applied in two 10‐min phases, the first phase at −15 mmHg and the second at −30 mmHg. Graded levels of LBNP were applied to assess “dose‐dependent” responses in both groups pre‐ and post‐FO supplementation.

### Cardiovascular measurements

Renal blood flow velocity (RBFV) was measured using Doppler ultrasound (HDI 5000; ATL Ultrasound, Bothell, WA) via the anterior abdominal approach (Momen et al. 2003; Wilson et al. 2007). The renal artery was imaged using a 2–5 MHz curved‐array transducer with a 2.5 MHz pulsed Doppler frequency. The probe was held in the same location for each trial, with an insonation angle of ≤60° to the renal artery, and the focal zone was set to the artery's depth. Because of the low Doppler ultrasound frequency required to measure RBFV and resultant limited spatial resolution, it is not possible to measure renal artery diameter and therefore calculate renal blood flow with this technique. However, it has been shown that the diameter of this large conduit vessel does not change significantly in response to pharmacological infusions that considerably alter renal blood flow (Marraccini et al. 1996; Manoharan et al. 2006). Therefore, changes in RBFV likely represent changes in renal blood flow. Beat‐to‐beat systolic, diastolic, and mean BP were measured with a photoplethysmographic finger cuff (Finometer; FMS, Arnhem, the Netherlands). Three BP measurements were taken at baseline with a semi‐automated upper arm cuff (Dinamap; GE Medical System; Milwaukee, WI), which were used to calibrate the baseline finger cuff signal in offline analysis. HR was measured with a three‐lead electrocardiogram (ECG, Cardiocap/5; GE Healthcare, Waukesha, WI). RBFV measurements were taken at baseline and during LBNP. BP and HR were measured continuously throughout baseline and LBNP.

### EPA and DHA quantification

The amount of EPA and DHA present in erythrocytes was quantified in a subset of young (*n *=* *7) and older (*n *=* *8) subjects before and after FO supplementation according to established laboratory methodologies (Harris et al. 2012b).

### Data and statistical analyses

Doppler images were analyzed using HDI 5000 software to produce beat‐to‐beat values for RBFV. RVR, an index of renal vasoconstriction (Momen et al. 2003; Wilson et al. 2007), was calculated as mean BP/RBFV. An analog‐to‐digital converter sampled data at 400 Hz, and data were displayed and recorded for offline analysis (MacLab 8e; AD Instruments; Castle Hill, NSW). Raw data files were analyzed to produce beat‐to‐beat values for systolic, diastolic, and mean BP, pulse pressure (PP), and HR. Absolute and relative (change from baseline) RVR, RBFV, mean BP, HR, systolic and diastolic BP, and PP values were calculated for the 3‐min baseline, the last 3 min of −15 mmHg LBNP, and the last 3 min of −30 mmHg LBNP in both the young and older groups for both visits. The data are shown as mean ± SEM.

Baseline differences in subject characteristics were assessed using a one within‐ (condition: pre‐ vs. post‐intervention) and one between‐ (age: young vs. older) factor, repeated measures analysis of variance (ANOVA). Differences in cardiovascular responses to LBNP were assessed using a two within‐ (condition: pre‐ vs. post‐intervention, and phase: −15 mmHg LBNP and −30 mmHg LBNP) and one between‐ (age: young vs. older) factor, repeated measures ANOVA. Post hoc analysis involving pairwise comparisons with a Holm‐Bonferroni correction was conducted when significant differences between factors were identified. Statistical significance was set at *P *<* *0.05, and all statistical analysis was performed using SPSS (IBM, Armonk, NY).

## Results

### Subject characteristics at rest

Baseline characteristics, cardiovascular, and EPA and DHA content, values of the young and older groups are shown in Table 1. RVR, RBFV, mean BP, systolic BP, PP, and HR at rest were similar in the two groups, but resting diastolic BP was higher in the older group than the young group (*P *<* *0.05). As expected, FO supplementation significantly increased EPA and DHA levels in both age groups (*P *<* *0.05). FO supplementation tended to increase DHA more (*P *=* *0.081), and EPA tended to be higher (*P *=* *0.085), in the older group compared to the young group. FO supplementation did not affect baseline cardiovascular values between visits. Baseline plasma creatinine and blood urea nitrogen levels were similar in both age groups, and were unaffected by FO supplementation.

### Effect of FO on renal responses to LBNP in young and older groups

**Figure 2:**
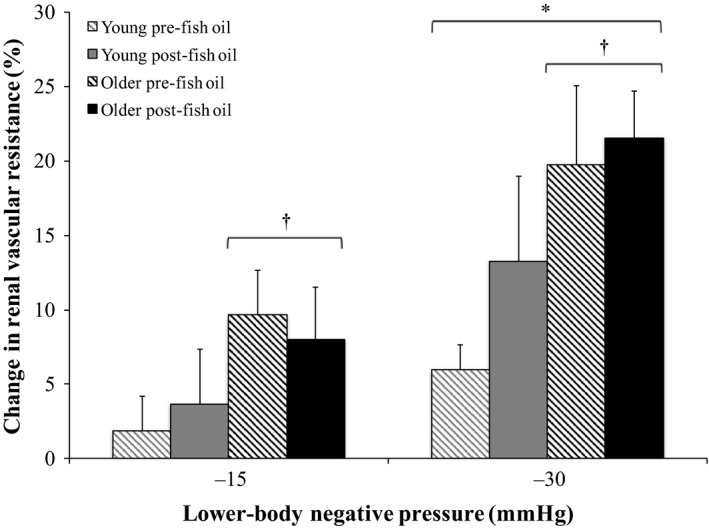
Relative changes from baseline in renal vascular resistance during graded lower‐body negative pressure (LBNP) pre‐ and post‐fish oil supplementation in the young (*n *=* *10) and older (*n *=* *10) groups (three‐way repeated measures ANOVA). *Significantly different from −15 mmHg LBNP (*P *<* *0.05). ^†^Significantly different from young (*P *<* *0.05).

**Figure 3:**
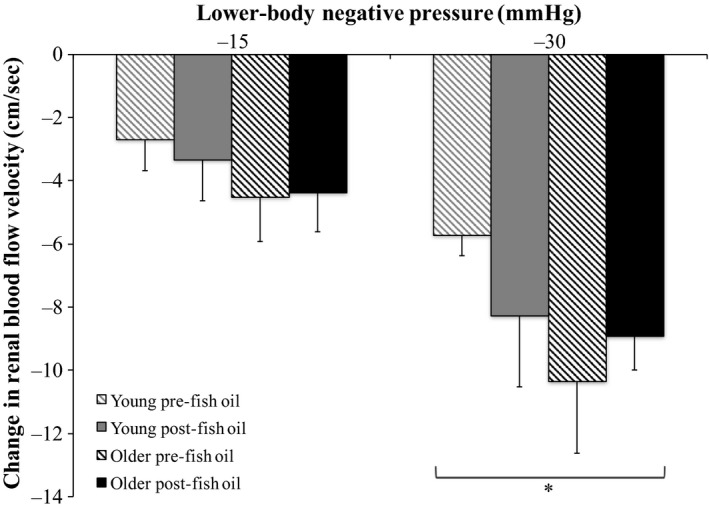
Relative changes from baseline in renal blood flow velocity during graded lower‐body negative pressure (LBNP) pre‐ and post‐fish oil supplementation in the young (*n *=* *10) and older (*n *=* *10) groups (three‐way repeated measures ANOVA). *Significantly different from −15 mmHg LBNP (*P *<* *0.05).

Relative changes from baseline in RVR and RBFV during graded LBNP pre‐ and post‐FO supplementation are shown in Figures 2 and 3, respectively. RVR increased (Fig. 2) and RBFV decreased (Fig. 3) in an intensity‐dependent fashion during LBNP in both the young and older groups (*P *<* *0.05). Additionally, RVR increases were greater in the older group compared to the young group (*P *<* *0.05). FO supplementation did not affect RVR or RBFV responses to graded LBNP in either the young or older group.

### Effect of FO on BP and HR responses to LBNP in young and older groups

**Figure 4:**
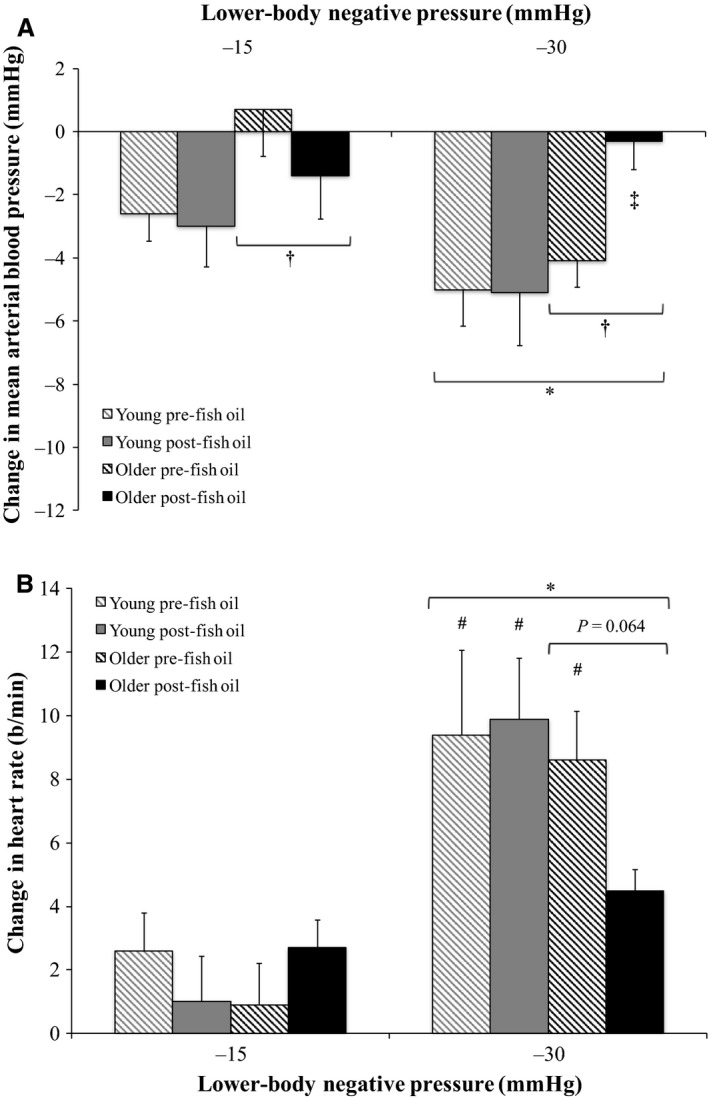
Relative changes from baseline in (A) mean arterial blood pressure and (B) heart rate during graded lower‐body negative pressure (LBNP) pre‐ and post‐fish oil supplementation in the young (*n *=* *10) and older (*n *=* *10) groups (three‐way repeated measures ANOVA). *Significantly different from −15 mmHg LBNP (*P *<* *0.05). ^†^Significantly different from young (*P *<* *0.05). ^‡^Significantly different from older −30 mmHg LBNP pre‐fish oil supplementation (*P *<* *0.05). ^#^Significantly different from −15 mmHg LBNP (from 3‐factor interaction; *P *<* *0.05).

**Figure 5:**
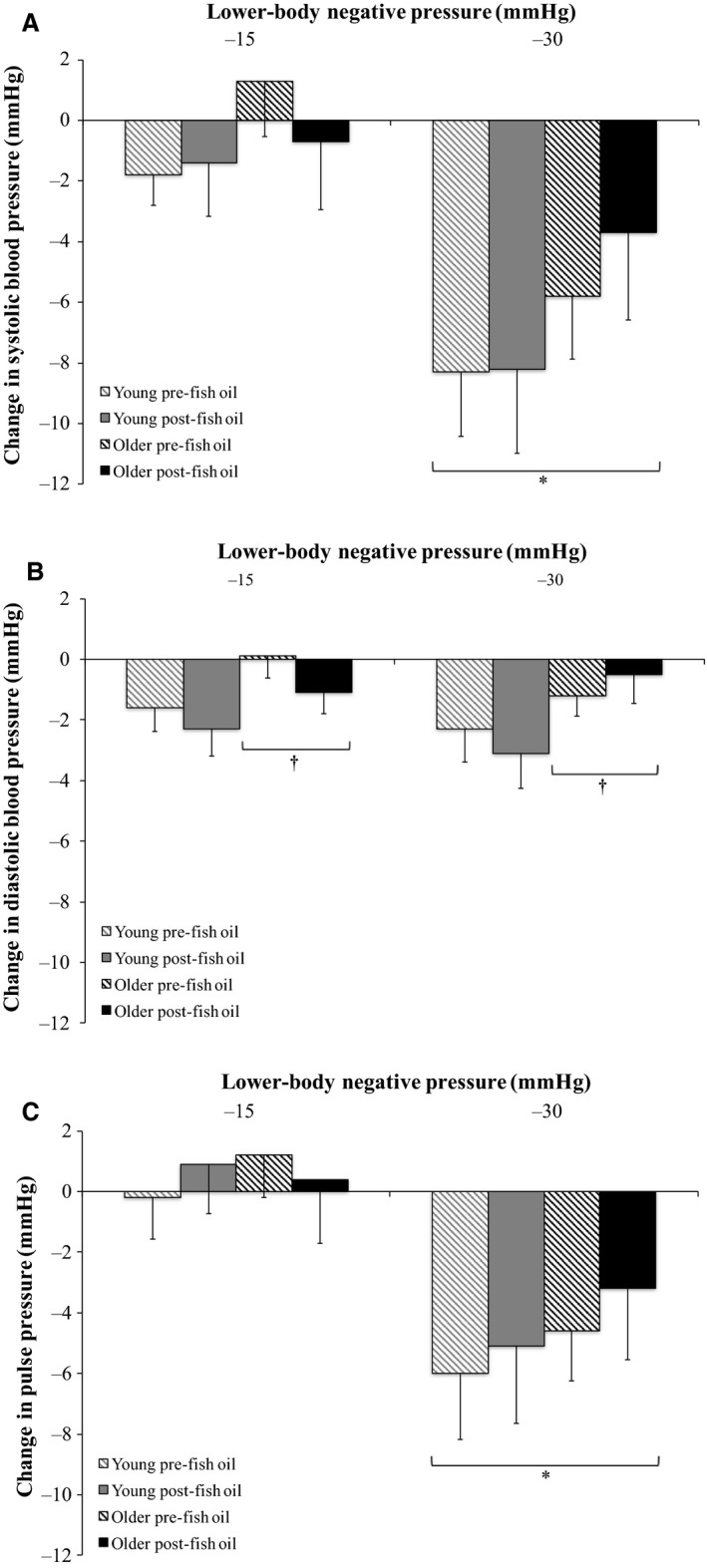
Relative changes from baseline in (A) systolic blood pressure, (B) diastolic blood pressure, and (C) pulse pressure during graded lower‐body negative pressure (LBNP) pre‐ and post‐fish oil supplementation in the young (*n *=* *10) and older (*n *=* *10) groups (three‐way repeated measures ANOVA). *Significantly different from −15 mmHg LBNP (*P *<* *0.05). ^†^Significantly different from young (*P *<* *0.05).

Relative changes from baseline in mean BP, HR, systolic BP, diastolic BP, and PP during graded LBNP pre‐ and post‐FO supplementation are shown in Figures 4 and 5. Mean BP (Fig. 4A), systolic BP (Fig. 5A), and PP (Fig. 5C) decreased and HR increased (Fig. 4B) in an intensity‐dependent fashion during LBNP in both the young and older groups (*P *<* *0.05). Diastolic BP did not change significantly with increasing LBNP intensity in either group (Fig. 5B), although decreases in diastolic BP were smaller in the older group compared to the young group across all trials (*P *<* *0.05). Mean BP decreases were also smaller in the older group compared to the young group across all trials (*P *<* *0.05). Additionally, FO supplementation significantly attenuated the mean BP decrease during −30 mmHg LBNP in the older group (*P *<* *0.05). This attenuated mean BP decrease corresponded with a smaller HR increase during −30 mmHg LBNP in the older group following FO supplementation. This reduced HR increase tended to be smaller than the HR increase during −30 mmHg LBNP in the older group prior to FO supplementation (*P *=* *0.064). In contrast, HR increases during −30 mmHg LBNP in the older group prior to FO supplementation and in the young group prior to and following FO supplementation were greater than during −15 mmHg LBNP.

## Discussion

The main findings from this study are that FO supplementation attenuates the mean BP response but does not affect the renal vasoconstrictor response to orthostatic stress in older adults. These findings suggest that omega‐3 polyunsaturated fatty acid consumption improves the maintenance of BP in response to orthostatic stress in older adults, without altering sympathetic outflow to the kidneys.

We recently showed that the renal vasoconstrictor response to orthostatic stress via LBNP is augmented in healthy older adults, while their BP response is similar to healthy young adults (Clark et al. 2015). As FO supplementation can attenuate sympathetic responses (Chin et al. 1993; Mori et al. 2000; Radaelli et al. 2006; Carter et al. 2013), we hypothesized that FO supplementation would reduce the greater renal vasoconstrictor response to LBNP in healthy older adults. Contrary to our hypothesis, our results indicate that FO supplementation does not affect RVR or RBFV responses to LBNP in either healthy older or young adults. However, FO supplementation did attenuate the mean BP response to the greatest level of LBNP applied in healthy older adults. These findings indicate that following FO supplementation, older adults still exhibit an exaggerated renal vasoconstrictor response to orthostatic stress, but their mean BP is better maintained at this time. This intriguing effect of FO supplementation suggests that omega‐3 polyunsaturated fatty acid consumption reduces orthostatic hypotension in healthy older adults, but an augmented renal vasoconstrictor response to orthostatic stress still occurs in these individuals. The heightened physiological response of augmented renal vasoconstriction during orthostatic stress in older adults remains acutely appropriate, as this greater reflex reduction in blood flow to the kidneys allows more blood volume to be held in the systemic circulation, and therefore BP can be better supported at that time. Therefore, it appears that following FO supplementation, BP can be more effectively maintained in the presence of this exaggerated renal vasoconstrictor response to orthostatic stress in older adults than without FO supplementation.

Although the objective of this study was not to identify the specific mechanism(s) involved, we can speculate an explanation for the observed FO‐induced reduction in orthostatic hypotension in older adults. As mean BP was better maintained (more similar to baseline) at −30 mmHg LBNP in the older group post‐FO supplementation (Fig. 4A), yet the older group still exhibited an augmented renal vasoconstrictor response to this level of LBNP that was unaffected by FO supplementation (Fig. 2), these divergent results suggest that the hypovolemic stimulus of LBNP was both sensed by cardiopulmonary baroreceptors and responded to similarly pre‐ and post‐FO supplementation, but changes in arterial BP detected by arterial baroreceptors were sensed and/or responded to more effectively post‐ compared to pre‐FO supplementation. LBNP caused venous pooling in the lower limbs, creating a hypovolemic stimulus that was sensed by cardiopulmonary baroreceptors and initiated an increase in sympathetic outflow to the kidneys, inducing renal vasoconstriction to shunt a greater volume of blood into the systemic circulation in an attempt to correct the hypovolemia present. These reflex response mechanisms appear to have functioned similarly during LBNP in the older group pre‐ and post‐FO supplementation. Conversely, as the hypovolemic stimulus of −30 mmHg LBNP decreased systolic BP (Fig. 5A) and PP (Fig. 5C), arterial baroreceptors would have detected these reductions in systolic BP and PP, and either sensed and/or responded to these changes more effectively in the older group post‐FO supplementation, as mean BP remained similar to baseline at this time, and the decrease in mean BP at −30 mmHg LBNP in the older group pre‐FO supplementation was prevented (Fig. 4A).

We recently showed in healthy older adults that FO supplementation decreases central arterial stiffness (Monahan et al. 2015), which supports this explanation of our current findings, as central arterial compliance is linked with age‐related differences in cardiovagal baroreflex sensitivity (Rowe 1987; Hunt et al. 2001; Monahan et al. 2001). In other words, FO supplementation may have decreased central arterial stiffness in the older group, thereby improving central arterial compliance, which would allow for greater mechanical distortion of arterial baroreceptors in the walls of the carotid sinuses and aortic arch. This capacity for greater mechanical distortion would consequently allow better detection of small(er) changes in systolic BP or PP, resulting in more effective buffering of arterial BP via corrective changes in cardiac vagal activity. In this case, only small corrections in HR would be needed to buffer and maintain arterial BP, as the greater sensitivity of detection of systolic BP or PP changes would lead to more effective corrections via changes in HR, as illustrated by the smaller HR increase in response to −30 mmHg LBNP in the older group post‐FO supplementation (Fig. 4B). Further study of arterial baroreflex sensitivity and overall arterial baroreflex function during LBNP in young and older populations pre‐ and post‐FO supplementation is needed to confirm this speculation. Regarding other possible mechanisms that could be involved in the observed effects of FO supplementation, decreased blood viscosity could play a role. Advancing age is associated with greater whole blood viscosity (Simmonds et al. 2013), and FO supplementation has been shown to decrease whole blood viscosity in patients with peripheral arterial disease aged 56–75 years (Woodcock et al. 1984). Lower blood viscosity would lead to less friction between blood and blood vessel walls and therefore improved circulation of blood volume, which would be advantageous during a hypovolemic stimulus such as LBNP, although further study would be needed to confirm this concept.

As aging is associated with a greater incidence of orthostatic hypotension (Rutan et al. 1992), which is linked with greater mortality (Masaki et al. 1998), there is a great clinical need for therapeutic interventions to address this significant issue. The primary implication of these findings is that as FO supplementation led to a better‐maintained mean BP during orthostatic stress in older adults, this effect may provide support towards the concept of increasing omega‐3 polyunsaturated fatty acid consumption in older adults to reduce instances of orthostatic hypotension. As omega‐3 polyunsaturated fatty acid consumption is known to be associated with lower cardiovascular mortality (GISSI‐Prevenzione Investigators, 1999; Predimed Investigators, 2016), modulation of this physiological mechanism via FO supplementation may be a contributing factor to this improvement in cardiovascular outcomes. Orthostatic hypotension is also associated with conditions such as Parkinson's disease, pure autonomic failure, and multiple system atrophy (Freeman 2008). Due to the FO‐induced reduction in orthostatic hypotension in older adults observed in our study, omega‐3 polyunsaturated fatty acid consumption could potentially provide this benefit as a therapeutic intervention in these specific patient groups. This could be in addition to other known benefits of omega‐3 polyunsaturated fatty acid consumption, particularly in Parkinson's disease (Bousquet et al. 2011). Overall, further investigations into the beneficial effects of omega‐3 polyunsaturated fatty acid consumption in these clinically significant areas are required.

Conversely, as older adults still exhibited an exaggerated renal vasoconstrictor response to orthostatic stress despite a FO‐induced reduction in orthostatic hypotension, repeated instances of orthostatic stress in older adults could still lead to cumulative decrements in renal function over time (Weinstein and Anderson 2010), with or without FO supplementation. Reductions in renal function are strongly linked to chronic kidney disease and its progression (Coresh et al. 2003), with chronic kidney disease becoming an increasing clinical burden, particularly due to its association with cardiovascular disease (Gansevoort et al. 2013). As chronic kidney disease is also linked with orthostatic stress (Franceschini et al. 2010), further efforts to understand the basic neurovascular mechanisms involved in the augmented renal vasoconstrictor response to orthostatic stress in older adults are critically needed.

One limitation of this study is the lack of a placebo control group. However, the within‐subject (pre‐ vs. post‐FO supplementation) and between‐group (young vs. older group) comparisons still provide novel evidence regarding the effect of FO supplementation on the renal vasoconstrictor response to orthostatic stress in healthy older and young adults. Also, some epidemiological studies have shown that erythrocyte EPA and DHA levels are higher with greater age (Sands et al. 2005; Harris et al. 2012a, 2013; Flock et al. 2013). Our data showing that baseline erythrocyte EPA and DHA levels were not different in the young and older groups could be due to the good health of our older subjects or having smaller sample sizes compared to epidemiological studies. Vandal et al. (2008) showed that FO supplementation of 1000 mg EPA and DHA daily for 3 weeks increased EPA and DHA levels in plasma total lipids in both young and older subjects of similar ages to those in our study. Therefore, it was expected that FO supplementation of the dosage and duration that we implemented would increase EPA and DHA levels in both age groups, as our dosage was similar to this (at least 900 mg EPA and DHA daily), and our duration was much longer (12 weeks). Furthermore, Vandal et al. (2008) showed that DHA levels increased more in their older subjects compared to their young subjects following their FO supplementation, with our findings showing the same trend. From reviewing the existing literature, it is not clear why our older subjects tended to have higher EPA and DHA levels than our young subjects, but one reason that has been proposed and received some discussion is decreased fatty acid metabolism (Sands et al. 2005; Harris et al. 2012a, 2013; Flock et al. 2013).

In summary, we have shown that FO supplementation attenuates the mean BP response but does not affect the renal vasoconstrictor response to orthostatic stress in older adults. These findings suggest that omega‐3 polyunsaturated fatty acid consumption improves the maintenance of BP in response to orthostatic stress in older adults, without altering sympathetic outflow to the kidneys.

## Conflict of Interest

No conflicts of interest, financial or otherwise, are declared by the authors.
